# Assessing Chikungunya risk in a metropolitan area of Argentina through satellite images and mathematical models

**DOI:** 10.1186/s12879-016-1348-y

**Published:** 2016-02-01

**Authors:** Diego Ruiz-Moreno

**Affiliations:** Grupo de Ecologia Computacional, Instituto de Ciencias Sociales y Administración, Universidad Nacional Arturo Jauretche, Florencio Varela, Buenos Aires Argentina

**Keywords:** Chikungunya, *Aedes albopictus*, Argentina, Epidemiological modeling, Habitat availability, remote sensing

## Abstract

**Background:**

Chikungunya fever is a viral disease that recently invaded the American continent. In America, it is transmitted mainly by the mosquito *Aedes aegypti,* but *Aedes albopictus* is the main vector in other regions of the world. This work estimates the risk of disease emergence and the corresponding population at risk for the case of a naive population in the metropolitan area of Buenos Aires, the capital city of Argentina.

**Methods:**

A classic metapopulation epidemiological model, that considers human and mosquito populations, was extended in order to include different environmental signals. First, the vital rates of the mosquitoes were affected by local temperature. Second, habitat availability estimated from satellite images was used to determine the carrying capacity for local mosquito populations. Disease invasion was proposed to occur at different moments of the year. For each scenario, Monte Carlo simulations were used to estimate the risk of disease invasion and the population at risk.

**Results:**

The risk of a Chikungunya outbreak displays strong temporal (seasonal) patterns as well as spatial variability at the level of neighborhoods in the study area. According to the model, Summer and Fall display high risk for a Chikungunya invasion. The population at risk displays less variation over the year underlying the importance of preventive actions.

**Conclusions:**

The ability of mapping habitat quality for vector-borne diseases allows developing risk analysis at scales that are easily manageable for public health officers. For this location, the correlation of disease risk with the season of the year and the habitat availability could provide information to develop efficient control strategies. This also underlines the importance of involving the whole community when developing control measures for Chikungunya fever and other recently invading vector-borne diseases such as Zika fever.

**Electronic supplementary material:**

The online version of this article (doi:10.1186/s12879-016-1348-y) contains supplementary material, which is available to authorized users.

## Background

Chikungunya fever is an emerging vector-borne infectious disease that recently invaded the Americas [1, 2]. This disease is caused by an alphavirus endemic to Africa and some regions in Southeast Asia [3]. In 1953, the Chikungunya Virus was first isolated in Tanzania [4] and at that time the mosquito *Ae. aegypti* appeared to be the main vector of the disease. However, during the last decade, Chikungunya virus reached naive human populations in several regions by hitchhiking on the expanding range of a different mosquito: the Asian tiger mosquito, *Ae. albopictus*. This mosquito species became an important vector for Chikungunya because of the result of one single mutation in the virus envelope that increased virus transmission 100 fold [5] and caused the first recent big epidemic in La Reunion island during 2005-6 [6, 7]. Soon after that, several outbreaks were reported in islands of the Indic Ocean followed by recurrent epidemics in India [8] and Southeast Asia [9, 10]. In 2007, Chikungunya cases were reported in Italy [11] and shortly after that the virus was detected in southern France [12]. Before long, an explosive epidemic started in the Americas, reaching at least 44 countries in a few months, with two (of the three) Chikungunya genotypes circulating [3, 13].

Symptoms are normally used to diagnose Chikungunya fever, and some misdiagnoses can occur because the first symptoms are very similar to those of dengue fever [14, 15]. A specific symptom for Chikungunya is a debilitating and prolonged joint pain, affecting the peripheral small joints [16]. Infected individuals additionally exhibit nonspecific symptoms, such as fever, severe joint pain, muscle pain, headache, nausea, fatigue and skin rash [17]. Mortality is rarely caused by Chikungunya, but it might often occur in patients with other health conditions [18, 19]. There is neither a licensed vaccine nor specific treatment for this disease, therefore, supportive care and isolation of infective individuals, as well as mosquito control, are the recommended actions once cases are detected in a community [20]. The development of vaccines is undergoing much research given the current expansion of the disease [21].

The incubation period for Chikungunya in humans (also known as intrinsic incubation period) is approximately four days. After that, some individuals develop symptoms and some became asymptomatic (with a 3:1 ratio). The infective stage normally lasts 7 days with a range from 4 to 11 days [16, 17]. It is believed that individuals acquire long-lasting immunity after the acute stage, although severe joint pain may persist for several months [16]. Mosquitoes feeding on infective individuals may be able to transmit the virus after the extrinsic incubation period that normally last 2 days (range 1–14 days) and remain infectious for their lifetime [16].

The role of *Ae. albopictus* as a vector for infectious diseases has been of concern during the last decades [22]. In the Americas, *Ae. albopictus* was introduced around 1987 to the US [23] and reached Argentina and Uruguay —at the southern end of the continent— in 1998 [24] and 2003 [25] respectively. Hence, the presence of the tiger mosquito in this continent provides a perfect backbone that might support the emergence and establishment of several vector-borne neglected infectious diseases including Chikungunya.

Until the availability of a commercially licensed vaccine for Chikungunya fever, the control of endemic and seasonal vector populations is the most effective strategy to reduce disease risk. These control strategies should take advantage of the fact that mosquito populations fluctuate seasonally in temperate areas mainly due to temperature effects and habitat availability. This work expands previous epidemiological models [26] to include habitat availability as well as local temperature to assess the risk of Chikungunya emergence in a temperate metropolitan area in Argentina. *Ae. albopictus* is the only vector considered in this modeling work due to its significant efficiency as a vector for Chikungunya fever. As in similar studies [27], a specific period in the year with high epidemiological risk is identified suggesting that actions to control vector population should be taken before that period. Additionally, the epidemiological risk is spatially disaggregated at the level of neighborhoods to help identify hot spots that may need thorough attention from public health officers.

## Methods

**Fig. 1:**
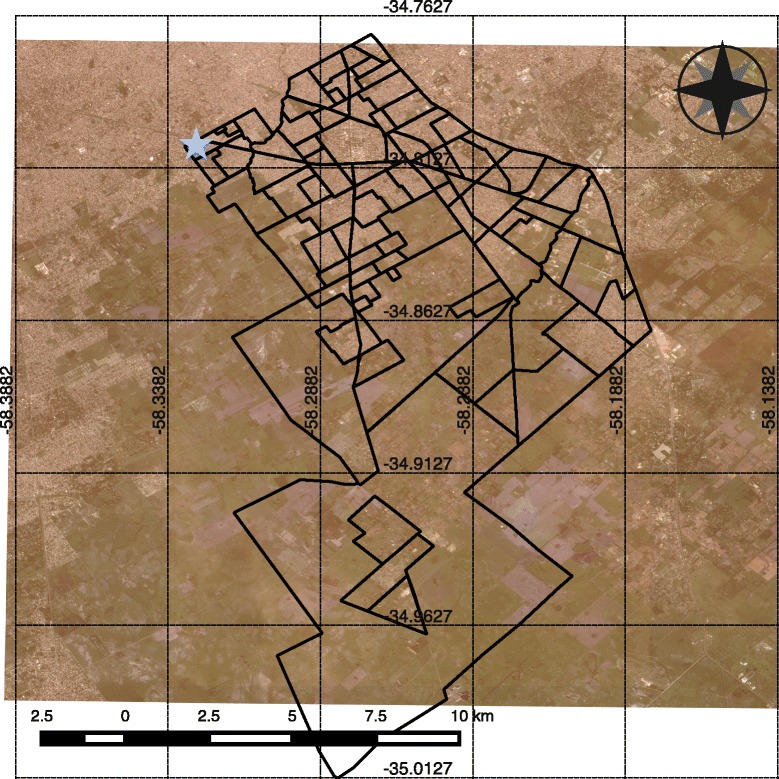
Map of the study area. The study area is composed of 94 neighborhoods. Background is a false color FORMOSAT-2 image. The star marks neighborhood #1 the site selected for disease introduction

The study area for this work was the Municipio of Florencio Varela, in the province of Buenos Aires, Argentina. This administrative unit expands over approximately 190Km^2^ and lies between 58° 21' W and 58° 10' W, and 34° 46' S and 35° 02' S (Fig. 1). With a population of 423,992 (census data 2010 [28]), this area is a part of the metropolitan area of the city of Buenos Aires, the capital city of Argentina, and it is politically divided into almost a hundred neighborhoods with different levels of urbanization ranging from urban to rural. This urbanization gradient correspond also with a biological, biodiversity, and socioeconomic gradients.

**Fig. 2:**
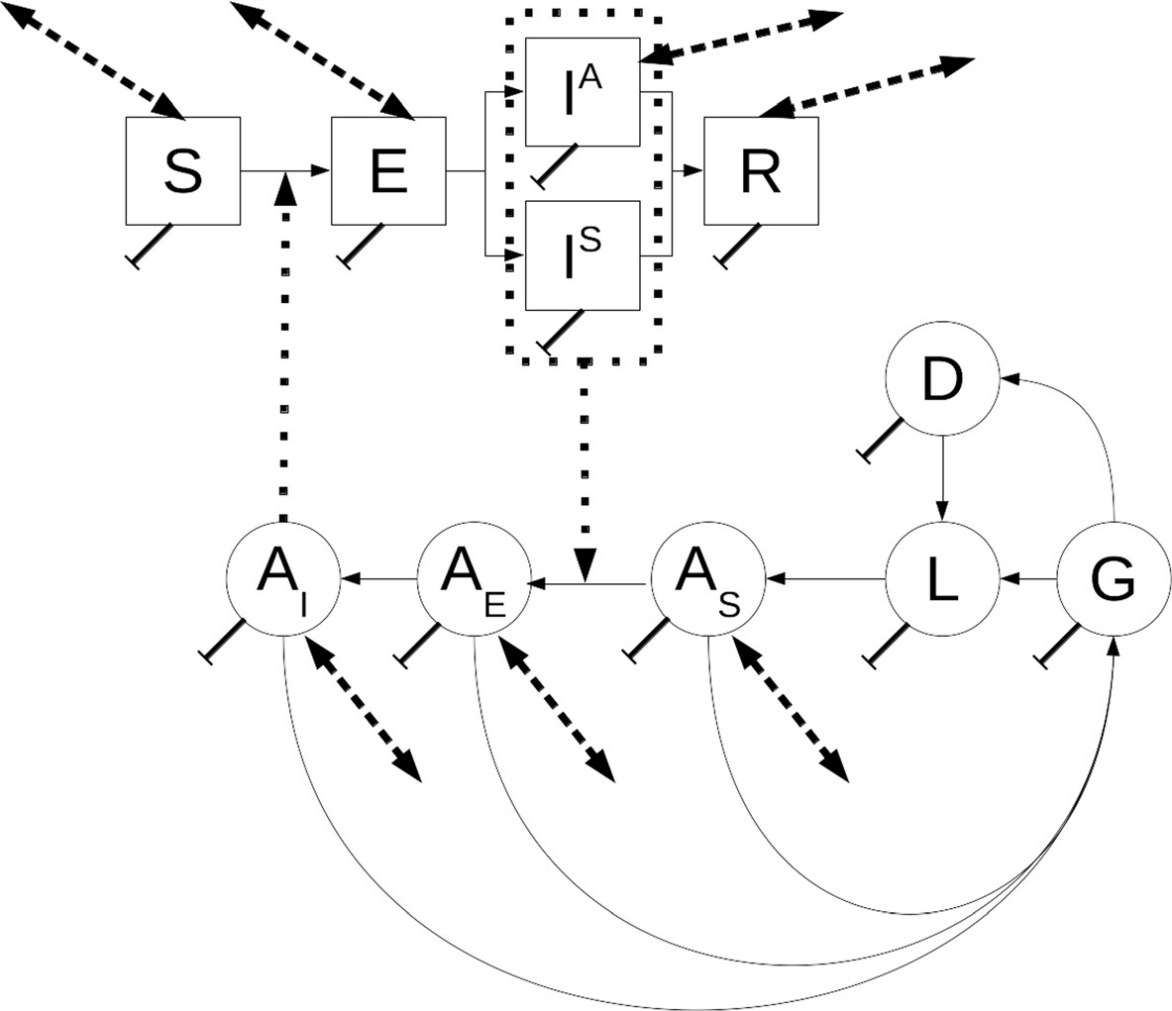
Graphical representation of the local population model. Boxes represent the human population, circles represent the mosquito population. At each neighborhood the human population is divided into (S) susceptible, (E) exposed, (I^A^) asymptomatic infected, (I^S^) symptomatic infected, and (R) recovered individuals. Similarly, the mosquito population are divided into immature stages, (G) eggs, (L) aquatic and (D) for eggs undergoing diapause, and (A_S_) susceptible, (A_E_) exposed and (A_I_) infective mature mosquitoes. Solid arrows represent transitions between these classes. Local population models for each neighborhood are linked by human commuting movement and mosquito migration patterns. Double arrowed dashed lines indicate the classes affected by movements of individual from and to analogous classes in other neighborhoods. Dotted lines describe disease transmission. Climate affects all the transitions in the mosquito populations. Habitat availability affects the coefficient of the density dependent term for immature stages of the mosquito populations. See details of the mathematical model in the Additional file 1

A classical epidemiological model that considers human and mosquito populations [29] was extended in order to include different environmental signals. At the core of this work, there is an epidemiological model that divides the human population into susceptible (S), exposed (E), symptomatic infective (I^S^), asymptomatic infective (I^A^) and recovered individuals (R). This model also considers the mosquito population to be divided into several categories: eggs (G), diapaused eggs (D) and aquatic (that comprehends larval and pupae developmental stages) (L) are the immature stages and susceptible (A_S_), exposed (A_E_) and infective (A_I_) are the adult stages (Fig. 2). Transitions between these categories describe the dynamics of this epidemiological system (see the mathematical formulation in the Additional file 1). Notice that an epidemiological model was implemented for each neighborhood in the study area. Thus, these 94 models were connected via movements of susceptible, exposed, asymptomatic infective and recovered human individuals, as well as for susceptible, exposed and infective mosquitoes, creating a full metapopulation epidemiological model.

At each neighborhood, the number of mosquito eggs (G) increases following a logistic curve, with parameters *f* for the adult fertility, and *ρ*_*M*_ for the strength of the density dependence. Mosquito eggs may be either affected by mortality at a rate *μ*_*G*_, undergo diapause at a rate *ζ*_*G*_ or mature into the aquatic stage at a rate *ξ*_*G*_. Notice that *μ*_*G*_, *ζ*_*G*_ and *ξ*_*G*_ depend on environmental temperature. In this model, eggs enter in a diapause state (D) at a rate *ζ*_*G*_ and leave that stage at a rate *ν*_*D*_. These two rates are a function of temperature. Here, the temperature is used as a proxy for daylight. The number of mosquitoes in the immature aquatic stage (L) increases by the maturation of eggs and diapaused eggs, with rates *ξ*_*G*_ and *ν*_*D*_ respectively. This aquatic stage includes both the larvae and the pupae developmental stages of the mosquitoes. Immature mosquitoes might suffer mortality at a rate *μ*_*L*_ or mature into adulthood at a rate *ξ*_*L*_. These two rates also are a function of environmental temperature. A density dependent effect (*ρ*_*A*_) controls the number of mosquitoes entering adulthood as susceptible adult mosquitoes (*A*_*S*_). In addition to that, susceptible adult mosquito population can decrease because temperature dependent mortality (${\mu}_{A_S}$) or because they become exposed to the disease with a force of infection that depends on the rate at which mosquitoes bite (*b*), the probability of disease transmission (*T*_*MH*_), and the proportion of infective humans (both symptomatic and asymptomatic, $\frac{I^S+{I}^A}{N_H}$). Exposed mosquitoes (*A*_*E*_) eventually become infective at a rate ${\sigma}_{A_E}$ or die at a rate ${\mu}_{A_E}$. Once again both rates depend on environmental temperature. Infective mosquitoes die at a temperature dependent rate ${\mu}_{A_I}$. Notice that adult mosquito population, either susceptible, exposed or infective, can increase because of the arrival of individual from other neighborhoods as described bellow. In the Additional file 1, the full formulation of the model as well as the specific functional forms for all the temperature dependent rates is presented. Parameter values are presented in Table 1.

**Table 1:** Parameter Values

Parameter	Value	Reference
*f*	4.4	[45]
*T*_*MH*_	0.977	[46–48]
*T*_*HM*_	0.726	[26, 49]
${\mu}_S={\mu}_E={\mu}_{I^S}={\mu}_{I^A}$	0.00003	This Work
*b*	$\frac{1}{3}$	[26, 49]
*σ*_*E*_	0.158	[50]
*c*	0.75	[26]
${\gamma}_{I^S}={\gamma}_{I^A}$	0.25	[51]
${\eta}_{I^S}={\eta}_{I^A}$	0.0001	[45]
*ρ*_*M*_	0.00001	This Work
*ρ*_*H*_	Varied by location to maintain a stable population size.	This Work

The human component of the model consist of susceptible individuals (S) that can increase as a function of natality (*ρ*_*S*_ is the strength of the density dependence that keeps population size constant) and can decrease because individuals become exposed to the disease. Exposition to disease is a function of the mosquito biting rate (*b*), the probability of disease transmission (*T*_*HM*_) and the ratio of infected mosquitoes respective to the human population ($\frac{A_I}{N_H}$). Exposed individuals (E) can either develop symptoms (*I* ^*S*^) or become asymptomatic infective individuals (*I* ^*A*^). Eventually, symptomatic and asymptomatic infective individuals recover from the disease (at equivalent rates ${\gamma}_{I^S}$ and ${\gamma}_{I^A}$, respectively). All human individuals can suffer natural mortality (with rates *μ*_*S*_ for susceptible, *μ*_*E*_ for exposed, ${\mu}_{I^A}$ for infective asymptomatic, ${\mu}_{I^S}$ for infective symptomatic, and *μ*_*R*_ for recovered) and for infective individuals (including both, symptomatic and asymptomatic), there is a small chance of suffering disease induced mortality at rates ${\eta}_{I^A}$ and ${\eta}_{I^S}$ respectively. All humans, but the symptomatic infective humans, are able to move between neighborhoods as described bellow.

In this metapopulation model, only a fraction of the individuals able to move was actually allowed to move between neighborhoods. The commuting rates for humans were set to be directly proportional to the population size, because bigger neighborhoods in the study area have more attractive commercial zones as well as administrative units, health-care facilities, and educational centers than smaller neighborhoods. The implementation of the human movements was such that as much as 50 % of the population was allowed to move, but these numbers were constrained to conserve the population size of the neighborhoods involved in the movement. These movements were included in the model in order to simulate the daily routine of susceptible adults (i.e., go to school, go to work, go to the market, etc.). Mosquito movement was restricted to a small fraction of the available population. Based on experimental data up to 4.53 % of the adult mosquito population was allowed to move from one location to another [30], however, no preferential direction was set. Mosquito migration rates were calculated assuming that individual mosquitoes do not flight more than 200 meters a day [31], hence only neighborhoods within that range were considered as destination of the migrant mosquitoes.

**Fig. 3:**
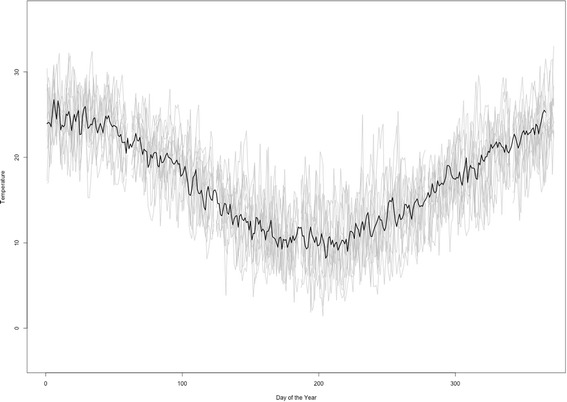
Temperature variation for the study region. Gray lines represent hourly temperatures for years 2004 to 2014. The black line, the mean of those values, was used as input for the mathematical model

All mosquito vital rates were affected by local temperature. A local typical temperature year was created by averaging ten years (2004–2014) of hourly temperature data obtained from NOAA for this region (Fig. 3). This typical temperature year was used as an input for the functional forms of the vital rates of the mosquitoes presented in the Additional file 1 [26].

**Fig. 4:**
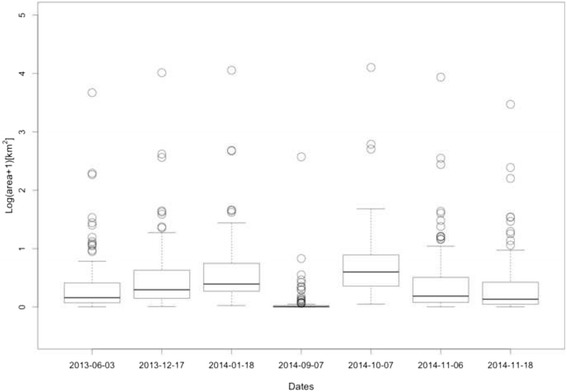
Estimated area for Mosquito Habitat. Each box plot represents the distribution for the area (in km^2^) available for immature mosquitoes at each neighborhood based on the wetness index. The dates correspond to the available satellite images

It is well known that mosquito population size is affected by several environmental and anthropogenic factors [32–34]. One factor of particular interest is habitat availability [35–37]. Given that *Ae. albopictus* is a peri-urban mosquito, there are few habitat constraints for adult mosquito when no control is implemented. Immature stages, however, have to develop in shallow-watered areas and therefore habitat availability could be more restrictive during that period. Usually, local rainfall records are used to estimate favorable conditions for mosquito development (for example, habitat availability). However, for this study area there are not official meteorological records that account for local precipitation data, and in addition to that the closest official precipitation station lies on a different river basin [38] and therefore rainfall records from that station may not be informative of mosquito habitat in the study area. Hence, high-resolution satellite images were used to determine habitat availability and consequently the carrying capacity for the immature mosquito populations. In 2014, through the ISPRS WG VI/5 research announcement FORMOSAT-2's satellite images were available for the study area. These images were used to evaluate mosquito habitat availability and its changes over the year. Seven images expanding over two years were used for this work. The dates of the images were: 2013-03-06, 2013-12-17, 2014-01-18, 2014-09-07, 2014-10-07, 2014-11-06, and 2014-11-18. A tasseled cap transformation [39, 40] was applied to each image. The tasseled cap transformation is a linear combination of the original satellite image bands that generates new bands that are almost orthogonal and represent “brightness” (relative high values representing little or no vegetation), “greenness” (relative high values represent vegetation) and “wetness” (relative high values represent damp land, among other things). For this study, the values of the wetness band (or index) were of particular interest. Only the cells that exhibited high values for the index wetness were considered appropriate as immature mosquito habitat. In the period 2013–2014 several haphazardly selected sites in the study area were examined to determine the presence of mosquito populations (regardless the species). The corresponding wetness digital values for these sites were recovered and used to determine a threshold value to map habitat quality. In this particular area, sites with a wetness value over that threshold (−50) were considered apt to harbor mosquito populations. Using this threshold value, it was possible to calculate the total area with appropriate immature mosquito habitat for each neighborhood in the study area (Fig. 4). A curve describing habitat availability at each moment of the year for each location was constructed by interpolating those values with a periodic spline. The values obtained with the spline were then used to estimate the carrying capacity for the immature stages of the mosquito population for each neighborhood (see Additional file 1 for details). Satellite images were processed with QGIS [41].

**Fig. 5:**
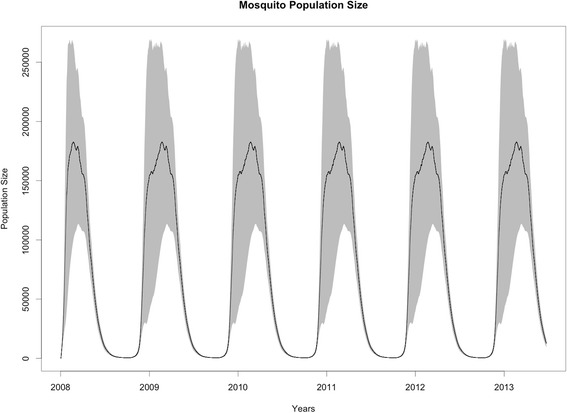
Mosquito population dynamics. The figure shows the mean and 95 % interval for the mosquito population size across neighborhoods

The model was implemented in C++ using a discrete time step (*Δt* = 0.1*days*) and discrete population sizes for both, humans and mosquitoes (i.e., the model keep track of the number of humans and mosquitoes in each of the stages described before). A Monte Carlo simulation approach was used to study the potential introduction of Chikungunya virus to the naive population of Florencio Varela, Buenos Aires, Argentina. Mosquito populations were simulated for several years, and once those populations reached dynamical equilibria the disease was introduced via one single exposed individual (Fig. 5 shows mosquito dynamics). Disease introduction was set to occur in neighborhood #1 (marked with a star in Fig. 1). This location has an average size and an average connectivity. A neighborhood that lies on the northern corner of the study area was used under the assumption that disease introduction will occur from the north where mosquito populations are better established and the two main points of entrance to the country are located (the main international airport and the main port). Changing the neighborhood of the disease introduction did not change significantly the results (results not shown). Twelve scenarios for introduction were used to evaluate the variation of epidemic risk along a year. A hundred replications were run for each scenario. The difference between the scenarios was the month of the introduction of the exposed individual, which was varied (monthly) from January to December. The disease was introduced the first day of the month. In each of the Monte Carlo replications, the model was simulated from identical initial conditions and the progression of the introduction of Chikungunya was analyzed.

**Fig. 6:**
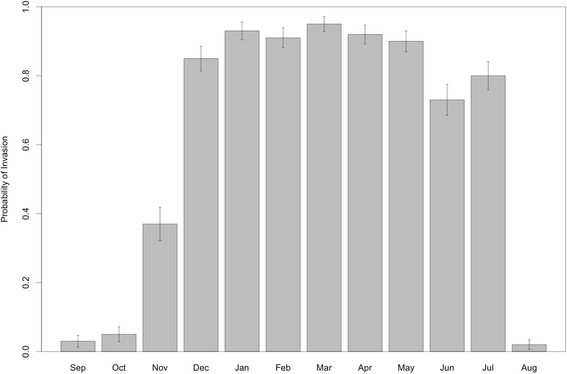
Probability of Invasion as a function of the month of disease introduction. The bars represent the probability of invasion (i.e., at least one transmission event occurred) calculated from the Monte Carlo simulations. Error bars are the standard error of the estimated mean probability

Several measures to quantify the results of the simulations of an introduction of Chikungunya in the study area were considered. First, the invasion probability was calculated by assessing whether or not a single transmission event occurred after the introduction. In addition to that, the probability of the occurrence of epidemics with a final size of 5 (small), 10 (medium) and 50 or more (big) individuals was calculated. Given the nature of the data (simulated data), it was possible to test whether a difference arises from considering these epidemic thresholds only considering symptomatic individuals or including asymptomatic individuals as well. For example, two values for the probability of a big outbreak were calculated. One when the final size of the epidemic was 50 or more cases but at least 50 of them were symptomatic cases (and hence this probability was based on the observed number of cases). The other value was calculated when at least 50 cases (including symptomatic and asymptomatic) were reached at the end of the outbreak. Furthermore, the epidemic risk was further characterized by using the peak value of disease incidence.

## Results and discussion

The risk of Chikungunya fever spreading over the naive population of Florencio Varela varied spatially and temporally over the year. According to the model the probability of an outbreak of Chikungunya fever changed from very low (3 % for September) to very high (95 % for March) as a function of the date of the introduction of the disease (Fig. 6). Similar values were obtained when different measures were used to estimate the probability of an outbreak (see Additional file 2: Figure S1). The lack of differences in the probability of observing outbreaks of final sizes 5, 10 and, 50 suggests that outbreaks are either relatively big or the introduction fails completely and the disease is unable to spread. This result should be pondered by the fact that in this modeling approach, the risk is homogeneous across all individuals inside each neighborhood, which might be an unrealistic assumption.

Despite the seasonality on the probability of invasion, the probability of an outbreak do not show a strong spatial component. It is possible to see that all neighborhoods display high values for this probability from December to June (summer and fall in this part of the world), and low values during the other parts of the year (winter and spring) (see Additional file 3: Figure S2). Lack of spatial heterogeneity follows from the fact that, in this model, human commuting patterns and mosquito movements might generate a strong diffusion of the disease after its introduction.

**Fig. 7:**
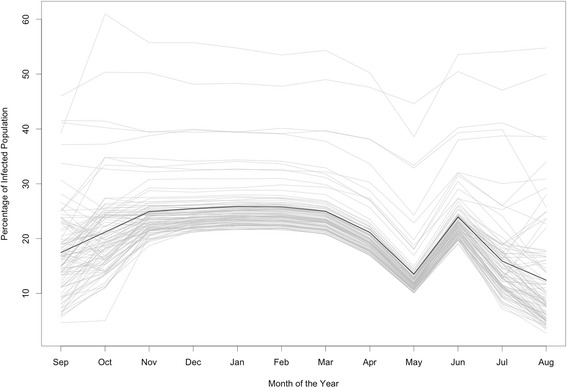
Mean percentage of infected population (counting symptomatic and asymptomatic individuals) as a function of the month of disease introduction. Gray lines represents individual neighborhoods. The black line is the arithmetic mean

When both symptomatic and asymptomatic are considered, the proportion of infectious individuals also displays seasonal variation, with higher values during the warmer months (later spring, summer and early fall) (Fig. 7). These seasonal patterns are not solely explained by temperature, but also due to the nonlinear response of the vectors to temperature variation (see Eq. 13–20 in the Additional file 1) and its, also non linear, interaction with habitat availability (see Eq. 21 in the Additional file 1). The sudden variation in the proportion of infected individuals at the end of the warm season (May-June) might be attributed to these interactions. Hence, it seems of particular interest to develop experiments for that range of temperatures and habitat availability. Seasonal variation is also particularly dependent on the model assumption of homogeneous mixing between symptomatic and asymptomatic at the level of neighborhood. However, it is important to notice that symptomatic individuals are likely to be detected by the health care system —in particular in Argentina where this service is free— and hence the number of asymptomatic individuals could potentially be estimated from those reports.

**Fig. 8:**
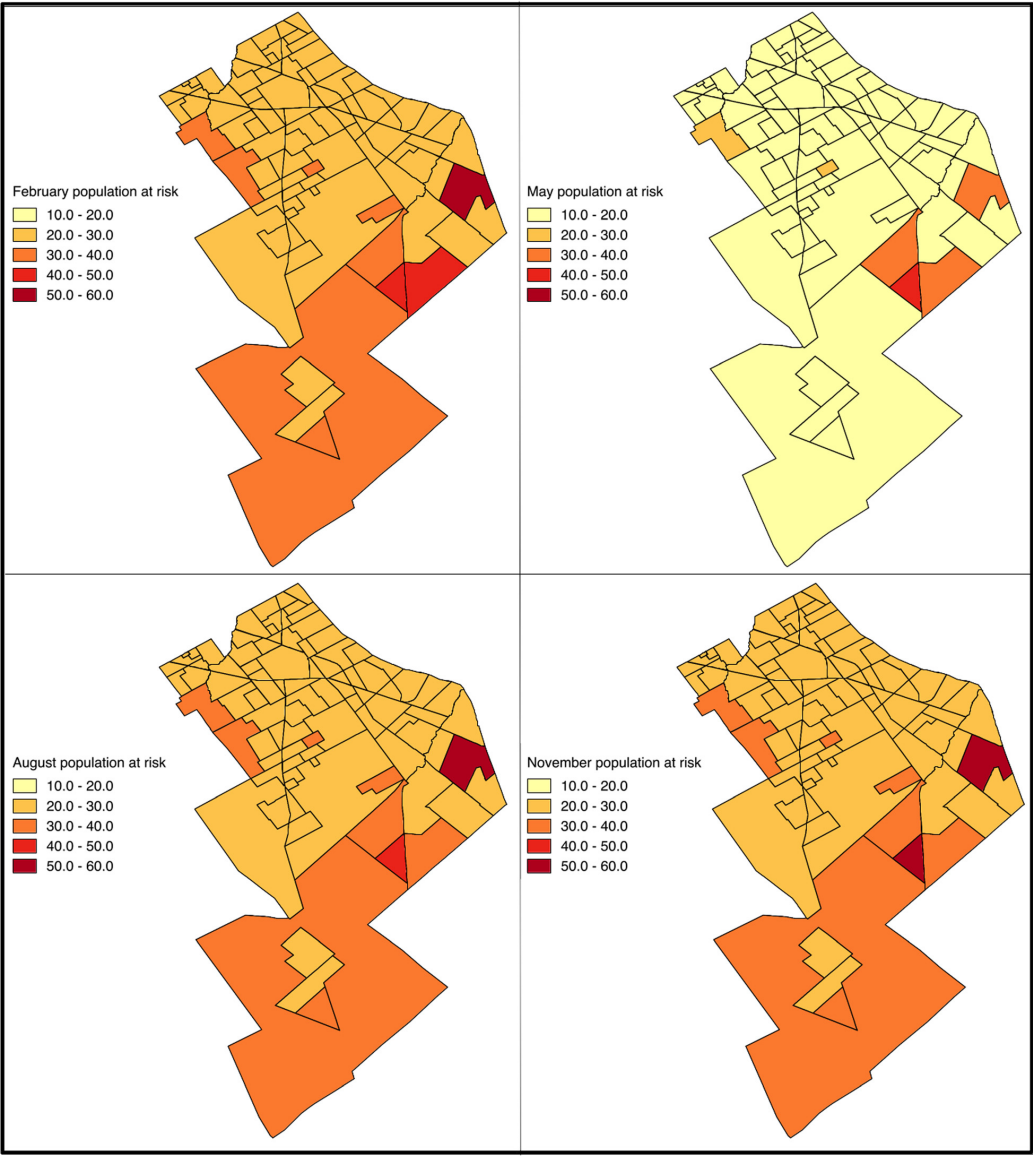
Percentage of the population infected at different moments of the year. The maps display for each neighborhood the mean percentage of the population that is infected (symptomatic plus asymptomatic) by Chikungunya from the Monte Carlo simulations. Colors darkens with increasing values

Spatial variability was also present in the mean size of outbreaks at each of the neighborhoods under study (Fig. 8). In this model, spatial variations in the force of infection can arise from the differences in the relative sizes of the populations of humans and mosquitoes. Therefore, it is expected that the variability in the outbreak size correlates with habitat availability, and thus individuals inhabiting some neighborhoods (high risk, red in Fig. 8) are consistently at more risk than those in others neighborhoods during the year.

## Conclusions

The model presented in this work suggests that Chikungunya fever could successfully invade Florencio Varela in Argentina. Given that the ongoing outbreak of Chikungunya fever in the Americas spread northwards and southwards from the initial cases in the Caribbean, it is possible for this disease to be imported via exposed individuals. In this modeling exercise, the consequences of importing only one individual exposed to the disease were studied. Despite the fact that this is an extremely conservative hypothesis, the probability of an outbreak after such event was high for some months of the year (i.e., December to June). These months correspond to Summer and Fall in Florencio Varela.

The findings presented in this work are in concordance with other works that show that a high-risk disease season is common not only for mosquito-borne [26, 42] diseases but also bacterial infections [43, 44]. The importance of the timing of disease introduction is a consequence of climatic covariates directly affecting the population dynamics of the vectors, the dynamics of the pathogens, and the transmission rate. In addition to temperature affecting vital rates of the mosquito and pathogen transmissibility, this work incorporated temporal and spatial variation in habitat availability for the vector. Incorporation of the habitat availability for this study area was done by using satellite images that allowed an indirect measure or estimation of the wetness in the landscape. Habitat availability constrained the population size of immature mosquitoes in the model presented in this work. Consequently, both the probability of an outbreak and the proportion of the population at risk were modulated by habitat availability.

In addition to environmental factors facilitating the establishment of the disease, in this model mosquito and human movements contributed to the spread of the disease from the initial point of introduction to all the neighborhoods. This underlines two —well known— important facts for disease prevention from the public health perspective: (1) reducing the movement of exposed individuals could reduce the spread of the disease and (2) reducing the population size of the vector could reduce the probability of disease transmission. This second point is central for several vector-borne diseases that have no specific treatment or commercial vaccines available, as is the case with Chikungunya, Dengue and Zika fever among others.

The use of satellite images allowed to spatially stratify the risk of disease emergence as well as to estimate the population at risk in the event of disease emergence. In order to better estimate these two indicators that are of interest for public health officers, additional information —some that might be derived from remote sensing— could also be included in the model presented in this work. For example, (a) house density and the degree of mixing between urban and rural environments could provide some information to estimate the level of exposition to mosquito populations; (b) rainfall data could be used to improve the mosquito population model as well as the dynamics of mosquito habitat availability; (c) spatially explicit thermal data could be also used to improve the mosquito population dynamics, (d) data on human movement and behavior in particular during the “mosquito season” could provide information to improve the naive hypothesis of contact between humans and mosquitoes at the local population level used in this work, and (e) actively screening mosquito population for virus presence could provide real time information of disease risk.

Although this model defines scenarios in which importation of one individual, that was exposed to Chikungunya, could lead to autochthonous transmission and outbreaks, these results were obtained for a specific set of parameter values (Table 1) and based on several assumptions. If we take into consideration that the mosquito *Ae. albopictus* displays strong plasticity (i.e., local adaptation) the obtained results could underestimate the output of an effective invasion. However, this model also does assume a naive reaction from the individuals and public officers, and hence might be overestimating the spread of the disease via mosquitoes and exposed individuals. In particular, this model does not consider the effects of (a) insecticide (at any level: individuals, household, neighborhood, the whole study area); (b) other biological actors that could partially exert control on mosquito population size, such as interspecific competition with other mosquito species, the effects of predators, etc.; (c) microclimate variability that could either affect the habitat availability as well as the survival of mosquitoes; and (d) heterogeneity in the ratio of mosquitoes to humans. Finally, better models for human movement could provide information that might lead to significant changes in the way that the disease spread in this area (This could be done, for example, by using information from mobile phones to develop and fit a detailed model for individual movement). Including these details into this model could be beneficial to study disease spread and the effects of potential actions to control disease spread. The objective of this work, however, was to quantify the potential for disease introduction in a naive area in the metropolitan area of Buenos Aires, the capital of Argentina. Concisely, the results presented here, generated under a conservative hypothesis for disease introduction and a naive reaction of the human population (i.e., no changes in behavior or use of control measures), strongly suggest that there is a high risk for an outbreak starting from one exposed traveler, and, therefore, we conclude that health systems in the region of the study area should be vigilant in order to have response systems ready.

The ability of mapping habitat quality for vector-borne diseases provides the unique opportunity of developing risk analysis at scales that are easily manageable for public health officers. In this work, the seasonal and spatial pattern of Chikungunya risk in a naive population was characterized. This opens the doors to the development of spatially explicit actionable points to prevent or mitigate —in the worse cases— the emergence of this terrible disease. For this location, in the southern limit of the distribution of *Ae. Aegypti*, disease risk appears to be highly seasonal and correlated with habitat availability. These two facts underline the importance of involving the whole population when developing control measures for Chikungunya disease and other recently invading vector-borne diseases such as Zika fever, because communities could help reducing habitat availability and report mosquito presence as well as suspicious cases.

## Additional files

Additional file 1: Text 1. Modeling details.

Additional file 2: Figure S1. Probability of disease invasion. Different colors represent different criteria. Red is based on one transmission event. Orange, a big outbreak (more than 50 individuals). Yellow, a medium outbreak74 (10 individuals). Green, a small outbreak (5 individuals). Pale colors (pale red, pale orange, pale yellow and pale green), are equivalent, but calculations only considered symptomatic individuals. (PNG 904 kb)

Additional file 3: Figure S2. Probability of disease invasion calculated as a single transmission event. Each line represents a neighborhood. (PNG 90 kb)
