# Supplementary material for: Assessing Chikungunya risk in a metropolitan area of Argentina through satellite images and mathematical models
Source: BMC Infect Dis. 2016 Feb 1;16:49. doi: 10.1186/s12879-016-1348-y (PMC4735964; doi:10.1186/s12879-016-1348-y)
Supplement: Supplementary file 1 — Modeling details. [file 12879_2016_1348_MOESM1_ESM.doc]

**Supporting Material for “Assessing Chikungunya risk in a metropolitan area of Argentina through satellite images and mathematical models”**

**D. Ruiz-Moreno**

**Mathematical Model**

The mathematical model presented in this work is a metapopulation climate driven epidemiological model. Hence, at the level of each local population (neighborhoods) the model presented graphically in Figure 2 controls the dynamics of mosquito and human populations. The populations are linked by human and mosquito movements between populations. Equations 1-12 describe the model for location $i$.

| $\frac{dG\left[i\right]}{dt}=f\cdot A_{M}\left[i\right]\cdot\text{e}^{-\rho_{M}\cdot A_{M}\left[i\right]}-\mu_{G}\cdot G\left[i\right]-\zeta_{G}\cdot G\left[i\right]-\xi_{G}\cdot G\left[i\right]$ | (Eq-1) |
| --- | --- |
| $\frac{dD\left[i\right]}{dt}=\zeta_{G}\cdot G\left[i\right]-\nu_{D}\cdot D\left[i\right]$ | (Eq-2) |
| $\frac{dL\left[i\right]}{dt}=\xi_{G}\cdot G\left[i\right]+\nu_{D}\cdot D\left[i\right]-\mu_{L}\cdot L\left[i\right]-\xi_{L}\cdot L\left[i\right]$ | (Eq-3) |
| $\frac{dA_{S}\left[i\right]}{dt}=\xi_{L}\cdot L\left[i\right]\cdot\text{e}^{-\rho_{A}\cdot A_{M}\left[i\right]}-\mu_{A_{S}}\cdot A_{S}\left[i\right]-\frac{b}{N_{H}\left[i\right]}\cdot T_{MH}\cdot\left(I^{S}\left[i\right]+I^{A}\left[i\right]\right)\cdot A_{S}\left[i\right]+\sum_{j=1}^{N} \phi_{ij}\cdot A_{S}\left[j\right]$ | (Eq-4) |
| $\frac{dA_{E}\left[i\right]}{dt}=\frac{b}{N_{H}\left[i\right]}\cdot T_{MH}\cdot\left(I^{S}\left[i\right]+I^{A}\left[i\right]\right)\cdot A_{S}\left[i\right]-\mu_{A_{E}}\cdot A_{E}\left[i\right]-\sigma_{A_{E}}\cdot A_{E}\left[i\right]+\sum_{j=1}^{N} \phi_{ij}\cdot A_{E}\left[j\right]$ | (Eq-5) |
| $\frac{dA_{I}\left[i\right]}{dt}=\sigma_{A_{E}}\cdot A_{E}\left[i\right]-\mu_{A_{I}}\cdot A_{I}\left[i\right]+\sum_{j=1}^{N} \phi_{ij}\cdot A_{I}\left[j\right]$ | (Eq-6) |
| $\frac{dS\left[i\right]}{dt}=N_{H}\left[i\right]\cdot\text{e}^{-\rho_{H}\cdot N_{H}\left[i\right]}-\mu_{S}\cdot S\left[i\right]-\frac{b}{N_{H}\left[i\right]}\cdot T_{HM}\cdot A_{I}\left[i\right]\cdot S\left[i\right]+\sum_{j=1}^{N} \kappa_{ij}\cdot S\left[j\right]$ | (Eq-7) |
| $\frac{dE\left[i\right]}{dt}=\frac{b}{N_{H}\left[i\right]}\cdot T_{HM}\cdot A_{I}\left[i\right]\cdot S\left[i\right]-\sigma_{E}\cdot E\left[i\right]-\mu_{E}\cdot E\left[i\right]+\sum_{j=1}^{N} \kappa_{ij}\cdot E\left[j\right]$ | (Eq-8) |
| $\frac{dI^{S}\left[i\right]}{dt}=c\cdot\sigma_{E}\cdot E\left[i\right]-\gamma_{I^{S}}\cdot I^{S}\left[i\right]-\mu_{I^{S}}\cdot I^{S}\left[i\right]-\eta_{I^{S}}\cdot I^{S}\left[i\right]$ | (Eq-9) |
| $\frac{dI^{A}\left[i\right]}{dt}=\left(1-c\right)\cdot\sigma_{E}\cdot E\left[i\right]-\gamma_{I^{A}}\cdot I^{A}\left[i\right]-\mu_{I^{A}}\cdot I^{A}\left[i\right]-\eta_{I^{A}}\cdot I^{A}\left[i\right]+\sum_{j=1}^{N} \kappa_{ij}\cdot I^{A}\left[j\right]$ | (Eq-10) |
| $\frac{dR\left[i\right]}{dt}=\gamma_{I^{A}}\cdot I^{A}\left[i\right]+\gamma_{I^{S}}\cdot I^{S}\left[i\right]-\mu_{R}\cdot R\left[i\right]+\sum_{j=1}^{N} \kappa_{ij}\cdot R\left[j\right]$ | (Eq-11) |
| $\frac{dW\left[i\right]}{dt}=\eta_{I^{A}}\cdot I^{A}\left[i\right]+\eta_{I^{S}}\cdot I^{S}\left[i\right]$ | (Eq-12) |

The increase in the number of mosquito eggs (Eq-1) is given by the fecundity$\left(f\right)$of the adult mosquito population$\left(A_{M}=A_{S}+A_{E}+A_{I}\right)$but controlled by the strength of the density dependence$\left(\rho_{M}\right)$. Mosquito eggs can suffer mortality ($\mu_{G}$which is a function that depends on temperature), undergo diapause ($\zeta_{G}$, also a function of temperature) or mature to an immature aquatic stage ($\xi_{G}$which depends on the temperature). In this model, mosquito eggs undergo diapause (Eq-2) in a simple manner, as a function of temperature eggs enter$\left(\zeta_{G}\right)$and leave$\left(\nu_{D}\right)$this stage. The immature aquatic stage of mosquitoes (Eq-3) is composed of the eggs that directly, or after diapause, matured. In this stage, the immature aquatic mosquitoes (i.e. larvae and pupae) suffer temperature dependent mortality$\left(\mu_{L}\right)$and temperature based maturation to adulthood$\left(\xi_{L}\right)$. With habitat availability determining the strength of a density dependence effect$\left(\rho_{A}\right)$, immature mosquitoes mature to susceptible adulthood (Eq-4). The adult susceptible population of mosquitoes suffers temperature based mortality$\left(\mu_{A_{S}}\right)$, and also can become exposed to the disease after biting (with a rate$b$, and a probability of disease transmission $T_{MH}$) the local infective human population$\left(I^{S}\left[i\right]+I^{A}\left[i\right]\right)$. Adult susceptible mosquitoes also move from one location to another as described by the matrix formed of coefficients$\phi_{ij}$. Exposed adult mosquitoes (Eq-5) may suffer temperature dependent mortality$\left(\mu_{A_{E}}\right)$or progress into the infective stage (with a temperature dependent rate,$\sigma_{A_{E}}$). Notice that exposed, as well as infected mosquitoes, move to neighboring areas with the same rates than the susceptible mosquitoes. In addition to movement, infective adult mosquitoes (Eq-6) also are affected by a temperature dependent mortality$\left(\mu_{A_{I}}\right)$. The model for the human population starts with a description of the susceptible individuals (Eq-7) that have a density dependent factor, to constrain the population size$\left(\rho_{H}\right)$, and a constant mortality rate$\left(\mu_{H}\right)$derived from demographic data [1]⁠. As with the mosquitoes, susceptible humans can become exposed to Chikungunya after being bitten by mosquitoes (at a rate$b$and with a probability of disease transmission$T_{HM}$). Finally, susceptible humans are allowed to move from one neighborhood to another following the commuting patterns described by the matrix formed by coefficients$\kappa_{ij}$. The exposed human individuals (Eq-8) become infective individuals at a constant rate$\left(\sigma_{E}\right)$and, as the case of the susceptible individuals, the number of exposed individuals changes because of commuting patterns and mortality$\left(\mu_{E}\right)$. A proportion$\left(c\right)$of the exposed individuals may develop symptoms during their infective period (Eq-9), and then either die naturally$\left(\mu_{I^{S}}\right)$or due to the disease$\left(\eta_{I^{S}}\right)$, or recover from the disease at a constant rate$\left(\gamma_{I^{S}}\right)$. Analogously asymptomatic infective individuals (Eq-10) may also suffer natural mortality$\left(\mu_{I^{A}}\right)$, disease induced mortality$\left(\eta_{I^{A}}\right)$or become fully recovered$\left(\gamma_{I^{A}}\right)$. Additionally asymptomatic individuals are allowed to commute. Recovered individuals (Eq-11) are able to commute to other neighborhoods and are only vulnerable to natural mortality$\left(\mu_{R}\right)$. One additional equation (Eq-12) was included in the model to account for the induced mortality of Chikungunya.

**Temperature dependent functional forms**

Several parameters of the equations 1-12 were considered to be a function of the environmental temperature. Experimental data were obtained and simple functional forms were fitted to those data [2]⁠.

| $\mu_{G}=\left\{\begin{matrix} 0 & temp\leq5 \\ 1-\frac{9.4-1.46\cdot temp+0.092\cdot temp^{2}}{100} & 5<temp\leq15 \\ 1-\frac{-167.9+11.74\cdot temp}{100} & 15<temp\leq20 \\ 1-\frac{-5.08+7.23\cdot temp-0.18\cdot temp^{2}}{100} & 20<temp\leq39.54 \\ 0 & 39.54<temp \end{matrix}\right\}$ | (13) |
| --- | --- |
| $\zeta_{G}=\left\{\begin{matrix} 1 & temp\leq0 \\ 1-0.1\cdot temp & 0\leq temp\leq10 \\ 0 & 10<temp \end{matrix}\right\}$ | (14) |
| $\xi_{G}=13.18-0.845\cdot temp+0.016\cdot temp^{2}$ | (15) |
| $\nu_{D}=\left\{\begin{matrix} 0 & temp<0 \\ \frac{temp}{10} & 0\leq temp\leq10 \\ 1 & 10<temp \end{matrix}\right\}$ | (16) |
| $\mu_{L}=\left\{\begin{matrix} 1 & temp\leq10 \\ 1-\frac{-136.08+17.8\cdot temp-0.37\cdot temp^{2}}{100} & 10<temp\leq38.5 \\ 1 & 38.5<temp \end{matrix}\right\}$ | (17) |
| $\xi_{L}=117.58-7.6\cdot temp+0.1321528\cdot temp^{2}$ | (18) |
| $\mu_{A_{S}}=\mu_{A_{E}}=\mu_{A_{I}}=\left\{\begin{matrix} 1 & temp<-5 \\ \frac{1}{5.6+1.12\cdot temp} & -5\leq temp<15 \\ \frac{1}{40.27-1.38\cdot temp+0.01\cdot temp^{2}} & 15\leq temp\leq38.5 \\ 1 & 38.5<temp \end{matrix}\right\}$ | (19) |
| $\sigma_{A_{E}}=\left\{\begin{matrix} \frac{1}{4} & temp<10 \\ \frac{22}{113-2.4\cdot temp} & 10\leq temp\leq32 \\ \frac{2}{3} & 32<temp \end{matrix}\right\}$ | (20) |

**Habitat Availability**

Habitat availability was calculated from the wetness layer obtained from applying a tasseled transformation [3, 4]⁠ to the satellite images. Equation 21 was derived to estimate the expected strength of the density dependence for the mosquito populations, based on the expected density of mosquitoes to humans.

| $\rho_{A}=\left(\frac{Area}{32.9562}\right)^{\frac{-1}{0.6664}}$ | (21) |
| --- | --- |

**Human movement details**

The human movement, as stated before, is controlled by the matrix$K$of coefficients$\kappa_{ij}$. Each coefficient is formed by the product of the amount of individuals allowed to move (that follows a binomial distribution with a mean that equals half of the population of the source neighborhood,$\frac{N_{i}}{2}$) times the attractiveness of the neighborhood of destination relative to the source neighborhood. The attractiveness of each neighborhood is defined in the following matrix

| Origen \ Destination | Big population size | Small population size |
| --- | --- | --- |
| Big population size | 5 | 0.1 |
| Small population size | 0.1 | 0.001 |

Notice that number of individuals moving between neighborhoods is algorithmically matched (i.e., the number of individuals moving from A to B equals the number of individuals moving from B to A) in order to keep the population size of neighborhoods invariant respect to movements.

**Parameter values**

**Figure Legends**

**Figure S1:** Probability of disease invasion. Different colors represent different criteria. Red is based on one transmission event. Orange, a big outbreak (more than 50 individuals). Yellow, a medium outbreak (10 individuals). Green, a small outbreak (5 individuals). Pale colors (pale red, pale orange, pale yellow and pale green), are equivalent, but calculations only considered symptomatic individuals.

**Figure S2:** Probability of disease invasion calculated as a single transmission event. Each line represents a neighborhood.

**References**

1. **Censo 2010** [http://www.censo2010.indec.gov.ar/]

2. Ruiz-Moreno D, Vargas I, Olson K, Harrington LC: **Modeling dynamic introduction of chikungunya virus in the United States**. *PLoS Negl Trop Dis* 2012, **6**:e1918.

3. Zeng ZY: **A new method of data transformation for satellite images: II. Transformation equations for SPOT, NOAA, IKONOS, Quick Bird, ASTER, MSS and other images and application**. *Int J Remote Sens* 2007, **28**:4125–4155.

4. Zeng ZY: **A new method of data transformation for satellite images: I. Methodology and transformation equations for TM images**. *Int J Remote Sens* 2007, **28**:4095–4124.
