# Supplementary figures and images for: Assessing Chikungunya risk in a metropolitan area of Argentina through satellite images and mathematical models
Source: BMC Infect Dis. 2016 Feb 1;16:49. doi: 10.1186/s12879-016-1348-y (PMC4735964; doi:10.1186/s12879-016-1348-y)

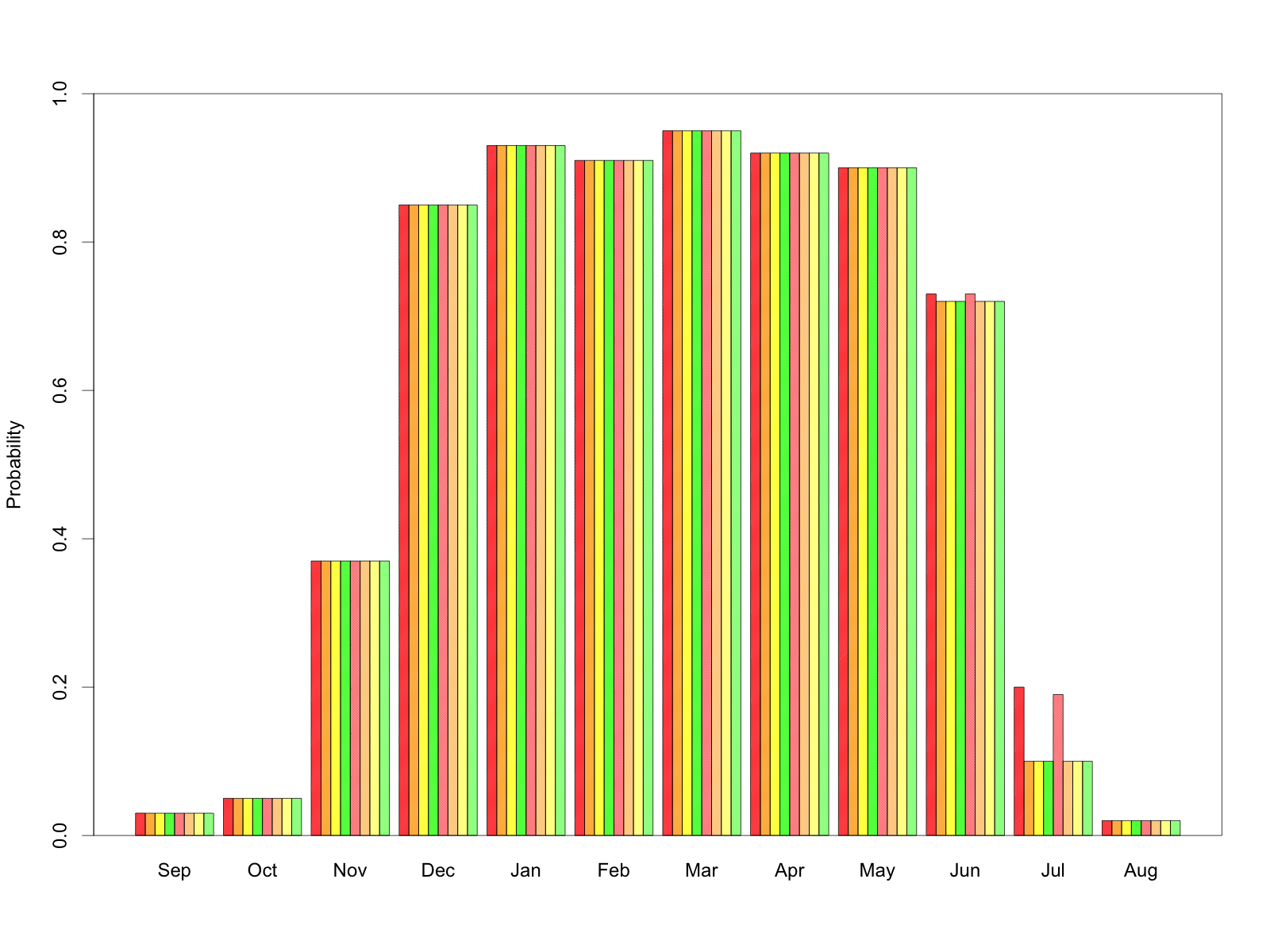

Supplement: Supplementary file 2 — Probability of disease invasion. Different colors represent different criteria. Red is based on one transmission event. Orange, a big outbreak (more than 50 individuals). Yellow, a medium outbreak74 (10 individuals). Green, a small outbreak (5 individuals). Pale colors (pale red, pale orange, pale yellow and pale green), are equivalent, but calculations only considered symptomatic individuals. (PNG 904 kb) [file 12879_2016_1348_MOESM2_ESM.png]

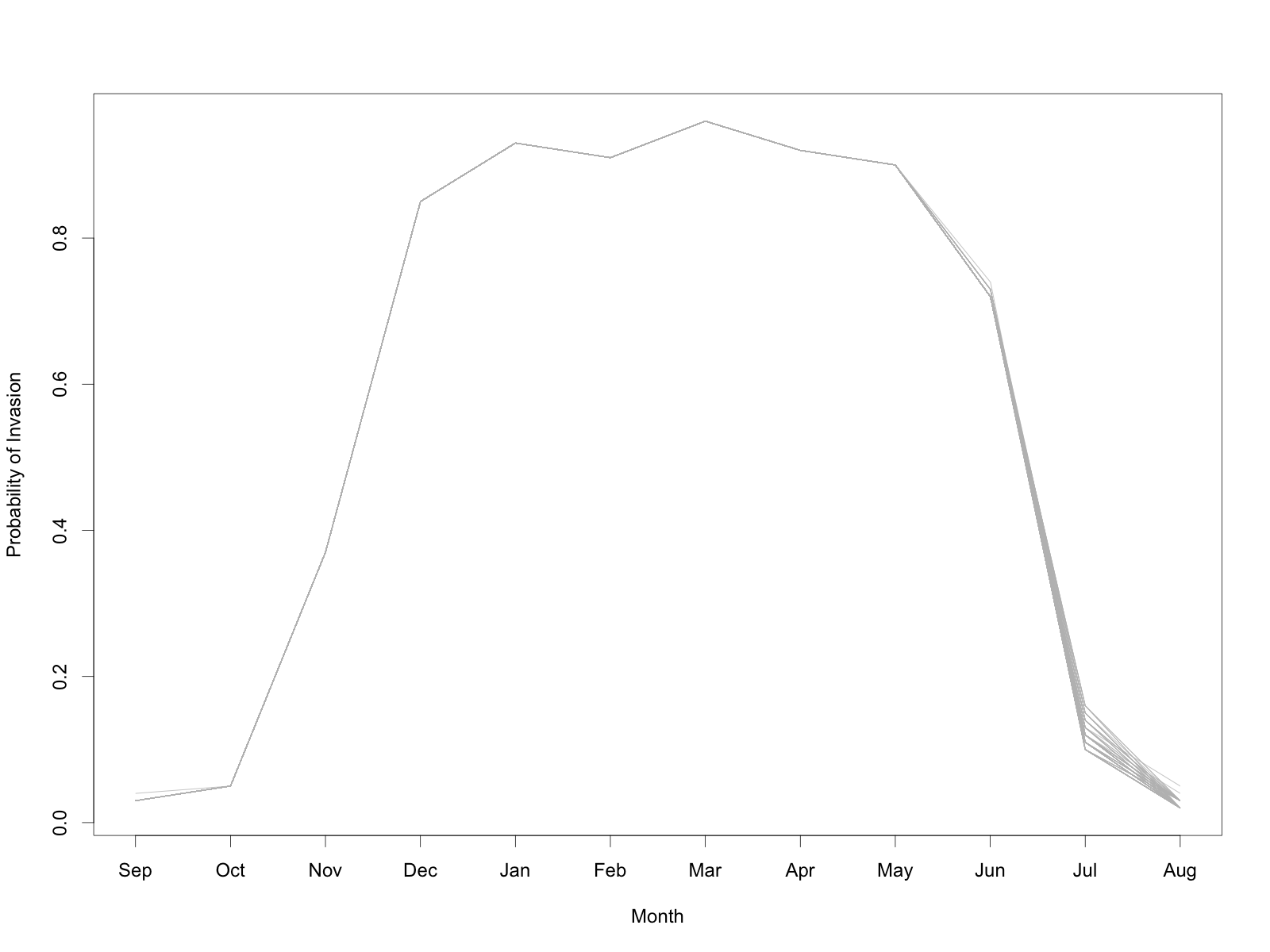

Supplement: Supplementary file 3 — Probability of disease invasion calculated as a single transmission event. Each line represents a neighborhood. (PNG 90 kb) [file 12879_2016_1348_MOESM3_ESM.png]
